# Intervention effects of telenursing based on M-O-A model in empty-nest older adult individuals with chronic diseases: a randomized controlled trial

**DOI:** 10.3389/fpubh.2024.1239445

**Published:** 2024-05-29

**Authors:** Yuan Yuan, Ping Hou, Sican Wang, Akio Kitayama, Kiyoko Yanagihara, Jingyan Liang

**Affiliations:** ^1^School of Nursing & School of Public Health, Yangzhou University, Yangzhou, China; ^2^Nagano College of Nursing, Komagane, Japan; ^3^Institute of Translational Medicine, Medical College, Yangzhou University, Yangzhou, China; ^4^Jiangsu Key Laboratory of Integrated Traditional Chinese and Western Medicine for Prevention and Treatment of Senile Diseases, Yangzhou University, Yangzhou, China

**Keywords:** telenursing, empty nest, older adults, chronic diseases, intervention effects

## Abstract

**Aim:**

This study aims to verify the effectiveness of M-O-A telenursing intervention model in improving the health status and quality of life of the empty-nest older adult individuals with chronic diseases by a randomized comparative trial.

**Methods:**

M-O-A telenursing intervention model was constructed based on the needs of the participants. The control group (*N* = 39) received routine nursing, the experimental group (*N* = 39) received M-O-A telenursing intervention in addition to routine nursing. After 12 weeks of intervention, the intervention effects of being a participant in the two groups were evaluated. SPSS 26.0 was used for data analysis.

**Results:**

After 12 weeks of intervention, for the experimental group, each dimension of quality of life based on EQ-5D-3L became better, especially for “pain/discomfort,” “anxiety/depression,” “HRQoL” and “EQ-VAS” (all *p* < 0.05) and each dimension of quality of life based on SF-36 became better too, especially for “GH,” “BP,” “RE,” “MH,” “VT,” “SF,” “PCS,” “MCS,” “SF-36” (all *p* < 0.05). In addition, there was a statistical downward trend in blood pressure, blood glucose, weight, BMI, fat rate, nap duration, number of nocturnal awakenings, light sleep rate and a statistical upward trend in water rate, basal metabolic rate, nighttime sleep duration, deep sleep rate, rapid eye movement sleep rate, especially at the end of intervention (all *p* < 0.05). While for the control group, there was no statistical improvement in all these aspects.

**Conclusion:**

The M-O-A telenursing model could effectively regulate quality of life and health condition of the empty-nest older adult individuals with chronic diseases, making it worthy of further promotion and application.

## 1 Introduction

With the improvement of economic level and the development of medical technology, the phenomenon of population aging is becoming more and more serious all over the world (1). In Japan, the older adults aged 65 and over comprised about 30% of the entire Japanese population now (2). In Italy, about 22.8% of the population is aged over 65 (3). In America, a predicted 20% of its entire population is forecast to be 65 years and over by 2030 (4). China is one of the world’s fastest-aging countries (5). It is estimated that China’s population aged 65 and over will reach the peak of the 21st century around 2058, with about 385 million people accounting for about 28.0% of the total population (6). With the transformation of the family life cycle, and the trend of independent living for children after marriage, the traditional family system’s position is weakened, more and more older adults people are becoming empty nesters (7, 8). The “empty nester” refers to the older adults (over 65 years of age) who live alone or with spouse, because they have no children or their children are married or work outside for a long time (9).

Population aging is the major attributing factor for most common chronic diseases (10). In China, nearly 90% of the aging population had a chronic disease by the end of 2018, according to China’s National Health Commission (11). “Health China 2030” released some key healthy indicators, for example, the life expectancy will increase by 3 years and reach to 79 years by 2030, the premature death rate caused by major chronic diseases will decline by 30%, and the proportion of personal health expenditure among the total health expenditure will decline by 25% (12). All of the indicators put forward an even greater need for the prevention and control of chronic diseases. Family support has been identified as an important beneficial effects for the older adults with chronic diseases (13). For empty-nest older adults individuals with chronic diseases, whether we can take measures to make up for the partial lack of family support is the concern of researchers. In recent years, more and more studies have pointed out that, telemedicine and telenursing interventions could possibly benefit them (14).

Telenursing is the use of technology to deliver nursing care and conduct nursing practice (14). The current telehealth trend shows that telenursing can significantly alleviate many medical difficulties by redesigning health-care practices and improving quality care delivery (15). Telenursing brings about impactful change that benefits both providers and clients by alleviating the time and costs for health care and improving access to care (16). The effects of telenursing services are significant and the acceptance rates among nurses and patients are very high. However, some studies have found that not all telenursing is superior to routine nursing, the main reason is the lack of effective assessment (17). Previous studies have found that empty-nest older adult individuals with chronic diseases have different demand degrees for different telenursing services (18). However, there is currently no research on providing corresponding telenursing based on patients’ needs and evaluating its effectiveness. Therefore, this study aims to build a telenursing intervention model based on previous research and to improve the health status and quality of life of empty-nest older adult individuals with chronic diseases.

## 2 Methods

### 2.1 Participants

This randomized controlled study was conducted Yangzhou and Nantong from January 1st to March 25th, 2022. Participants were eligible if they met the following inclusion criteria: (1) age ≥ 65 years; (2) with at least one chronic disease (including hypertension, hyperlipemia, diabetes, osteoarthropathy, gastrointestinal diseases, respiratory diseases, cerebral vascular diseases, heart disease, cancer and so on); (3) living alone or with their spouses, not living with their children; (4) living in the investigated community for more than 12 months, that is, the permanent resident population of the community; (5) having smart phones or other devices that can receive telenursing information; and (6) participating in the survey voluntarily. Participants were excluded if they met any exclusion criteria: (1) in the acute stage of disease, or severe cardiopulmonary, renal insufficiency, or terminal disease stage; (2) with communication, cognitive, or mental disorders; (3) being participating in other research. Previous studies reported chronic diseases situation among older adults people in communities is relatively complex, many people have multiple types of chronic diseases, and the severity of different chronic diseases varies (19). Therefore, this study did not distinguish disease types or severity. Written informed consents were obtained from all participants and they could withdraw from the study at any time.

### 2.2 Sample size calculation

According to the literature reports (20), the minimum sample size is calculated by the following formula: $n_{1}=n_{2}=2{\left[\left(u_{\alpha }+u_{\beta }\right)/\left(\delta /\sigma \right)\right]}^{2}+1/4\;{u_{\alpha }}^{2}$ . *α* = 0.05 and *β* = 0.10 and δ/σ = 0.80, so n_1_ = n_2_ = 34. To account for possible attrition, the sample sizes of the experimental group and the control group were increased by 20% to 40.

### 2.3 Participation process

Based on our previous study (18), researchers randomly selected one rural and one urban community in Nantong and Yangzhou, respectively. Researchers used the RAND function in Microsoft Excel to randomly select (21) 10 Arabic numbers (corresponding to participants’ house numbers) in each community as control group and 10 Arabic numbers (corresponding to participants’ house numbers) in each community as experimental group. Because all 80 of these older adults were informed and consented the research in our previous step of the study (18), all of them were willing to participate in this step of the study. In the experimental group, one participant from rural community in Nantong withdrew and in the control group, one participant from rural community in Yangzhou withdrew. Finally, 39 participants in the experimental group and 39 participants in the control group were included.

### 2.4 Survey tools

#### 2.4.1 EuroQol five-dimensions three-level questionnaire (EQ-5D-3L)

The EQ-5D-3L is a standardized health-related quality of life assessment tool first proposed by the EuroQol Group, which consists of two essential parts: a multidimensional health descriptive system and the visual analog scale (VAS). The EQ-5D descriptive system comprises five dimensions: mobility (MO), self-care (SC), usual activities (UA), pain/discomfort (PD) and anxiety/depression (AD). The Chinese version of the EQ-5D-3L has passed reliability and validity tests (22) and the formula for calculating an individual’s health-related quality of life (HRQoL) is as follows: HRQoL = 1–0.0274-0.0727* MO2-0.1788* MO3-0.1105* SC2-0.2349* SC3-0.0880* UA2-0.1518* UA3-0.0898* PD2-0.1667* PD3-0.0851* AD2-0.1702* AD3. The higher the EQ-5D-3L utility score, the higher the overall quality of life of the individual.

#### 2.4.2 MOS 36-item short form health survey (SF-36)

The SF-36, translated by the School of Medicine, Zhejiang University, includes 36 items. The higher the score, the better the quality of life. The scale consists of nine dimensions: general health (GH), physiological function (PF), role physiological (RP), body pain (BP), role emotional (RE), mental health (MH), vitality (VT), social function (SF) and health transition (HT). The scale can be divided into two subscales: the physical component summary (PCS) and the mental component summary (MCS). The Cronbach’s alpha values for the internal consistency reliability were 0.72 and 0.88, respectively. The 2-week test–retest reliability coefficients were 0.66 and 0.94, respectively (23). The MCS includes VT, SF, RE, and MH. The PCS includes PF, RP, BP, and GH. The total score on the SF-36 is the sum of all items except HT.

### 2.5 Intervention methods

#### 2.5.1 The control group received routine nursing

Community nursing staff carried out routine nursing in the control group without researchers’ interference. According to China’s National Basic Public Health Service, community nursing staff provide health management services for each older adult individual once a year. Annual health management services may or may not be provided during the study period.

#### 2.5.2 The experimental group received routine nursing and the M-O-A telenursing

**Figure 1:**
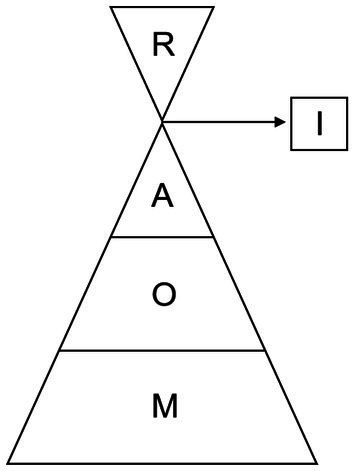
The M-O-A model.

Based on the Kano model proposed by a Japanese professor named Kano and the results of our previous study (18), researchers established the M-O-A telenursing intervention model, as shown in Figure 1. The model is shaped like a funnel, from the bottom up according to the proportion. M (Must-be qualities) means we should provide services at any time. O (One-dimensional qualities) means we should provide services regularly. A (Attractive qualities) means we should provide services irregularly. I (Indifferent qualities) means we should provide services when necessary. R (Reverse qualities) means we should not provide services at all. Almost all the measures were led by nurses. Disease screening and diagnosis were performed by doctors after nurses collect relevant data. Life cares, such as turning over and patting on the back, were performed by nurse workers. Specific measures are as follows:

① M telenursing measures.

M1: Remote one-button emergency caller: When the participants encountered any emergency, they could call the researchers through the remote one-button emergency caller (JIANTAOKJ, China), and the researchers would provide assistance at any time according to the situation of them.

M2: Remote emergency assistance arrangement: When the participants had a sudden disease, the researchers arranged corresponding green channels for them according to the priority of the disease to realize remote early triage before medical treatment.

M3: Remote monitoring of vital signs and sleep: Through the smart bracelet (Huawei, Band 6, China), the vital signs and sleep conditions of the participants were monitored. Once the vital signs of the participants were abnormal, the researcher would receive the signal at the first time and dealt with it accordingly.

② O telenursing measures.

O1: Regular family visits: Once 2 weeks, the researchers conducted regular family visits to the participants to implement health management services for patients.

O2: Remote diagnosis of diseases: Before the intervention, the health data of the participants were entered into the system. The researchers dynamically analyzed the relevant data, and remote disease diagnosis was implemented for the participants.

O3: Remote health counseling: Remote health counseling was open once a day, the participants could ask researchers any health questions.

O4: Remote rehabilitation guidance: Every Thursday, researchers gave remote rehabilitation guidance to the participants. First, they gave the participants a sensory understanding by micro classes and small videos, then the participants could have a video call with the researchers.

O5: Remote return visits and related health education: Every Saturday, the researchers provided return visits and related health education for the participants.

③ A telenursing measures.

A1: Remote education on home safety prevention: The researchers provided remote education on home safety prevention for the participants from time to time by micro classes and small videos, such as how to prevent falls and fraud.

A2: Teletraining on care skills: The researchers provided teletraining on care skills from time to time. For example, researchers taught them to master mental and physical care skills and the skills of mutual assistance among family members and community residents.

A3: Remote lectures about disease prevention: The researchers had remote lectures from time to time to teach the knowledge of disease prevention for common chronic diseases, unusual chronic diseases, acute diseases, infectious diseases and so on.

A4: Remote screening for diseases: Researchers carried out disease screening for the participants. According to the health data of the participants entered into the system, researchers screened for high-risk factors and judged various diseases.

A5: Distance intervention for disease risk factors: Researchers have evaluated and intervened in disease risk factors for the participants from time to time.

④ I telenursing measures.

I1: Remote calls for life needs: If necessary, the participants could put forward the demand for life care services to the researchers, and the researchers would contact the corresponding staff to provide door-to-door services such as turning over and patting their backs, washing their hair and face, trimming their nails and so on.

I2: Remote calls for nursing needs: If necessary, the participants could put forward nursing service demands to researchers, and the researchers would contact the corresponding staff to provide door-to-door services such as pressure injury care, wound stoma care, diabetes foot care and so on.

#### 2.5.3 Observation index

1. Participants needed to complete the questionnaires. Before intervention, researchers instructed participants to fill in the sociodemographic characteristics questionnaire, EQ-5D-3L and SF-36. After 12 weeks of intervention, all the participants completed the EQ-5D-3L and SF-36 again.
2. Before the intervention and at the end of the 2nd, 4th, 6th, 8th, 10th, and 12th week of the intervention, researchers measured the participants’ blood pressure and blood glucose, and used body fat scales (Huawei, HEM-B19, China) to monitor participants’ weight, BMI, fat rate, water rate, protein rate, basal metabolic rate. Additionally, researchers used smart bracelets to monitor participants’ sleep, such as light sleep, deep sleep, and rapid eye movement (REM).

#### 2.5.4 Analysis of data

Data were analyzed with IBM SPSS Statistics 26.0 software. If the measurement data conformed to a normal distribution, they were described as the mean ± SD and compared by t-test. If the measurement data did not conform to a normal distribution, they were described with the median and quartile (M, P25/P75) and compared by a nonparametric test. Mann–Whitney U test was used to examine the differences between experimental group and control group. While Wilcoxon test was used to examine the differences between before and after intervention in the same group. The counting data were described as frequencies and percentages, which were compared by the chi square test or rank sum test. All statistical tests were two-sided, and *p* values less than 0.05 were considered statistically significant.

#### 2.5.5 Control of bias

A study may have selection bias, information bias, and confounding bias. This study used random sampling, strictly controlled inclusion and exclusion criteria, to tried to ensure there was no statistically significant difference in baseline data between the experimental group and the control group. Participants were fully informed before the study, which improved their understanding and compliance with the study to ensure both groups of participants had low dropout rates. In addition, all researchers have undergone homogenization training to ensure consistency of the intervention. Two data analysts were blinded to the grouping, and statistical experts have been consulted for the selection of statistical methods to ensure the identification and avoidance of confounding factors. This study minimized bias as much as possible through the above methods.

#### 2.5.6 Ethical approval

The study was reviewed and approved by the Ethics Committee of School of Nursing, Yangzhou University (YZUHL2019001). Written informed consents were obtained from all participants. All personal information was encrypted.

## 3 Results

### 3.1 Baseline data between the experimental group and the control group

39 participants in the experimental group and 39 participants in the control group were included in this study. As shown in Table 1, there were no statistically significant in the baseline data between the two groups (all *p* > 0.05).

**Table 1:** Baseline data between the experimental group and the control group.

	Experimental group N(%)	Control group N(%)	*x*^2^/*t*	*p*
Gender				
Female	17 (43.59)	14 (35.90)	0.48	0.488
Male	22 (56.41)	25 (64.10)		
Age (years old)	72.87 ± 5.02	71.51 ± 4.42	1.27	0.209
Education level				
Junior high school and below	18 (46.15)	20 (51.28)	0.21	0.651
Senior high school and above	21 (53.85)	19 (48.72)		
Living alone				
Yes	17 (43.59)	23 (58.97)	1.85	0.174
No	22 (56.41)	16 (41.03)		
Number of chronic diseases				
≤ 2	15 (38.46)	13 (33.33)	0.22	0.637
>2	24 (61.54)	26 (66.67)		
Residence				
Urban residents (Yangzhou)	10 (25.64)	10 (25.64)	0.11	0.991
Urban residents (Nantong)	10 (25.64)	10 (25.64)		
Rural residents (Yangzhou)	10 (25.64)	9 (23.08)		
Rural residents (Nantong)	9 (23.08)	10 (25.64)		
Number of patients				
Hypertension	25 (64.10)	27 (69.23)	0.23	0.631
Osteoarthropathy	17 (43.59)	15 (38.46)	0.21	0.645
Hyperlipemia	12 (30.77)	12 (30.77)	0.00	1.000
Gastrointestinal diseases	9 (23.08)	12 (30.77)	0.59	0.444
Respiratory diseases	10 (25.64)	11 (28.21)	0.07	0.799
Cerebral vascular diseases	11 (28.21)	9 (23.08)	0.27	0.604
Heart disease	7 (17.95)	9 (23.08)	0.32	0.575
Diabetes	8 (20.51)	9 (23.08)	0.08	0.784
Cancer	3 (7.69)	2 (5.13)	0.21	0.644
Others	10 (25.64)	11 (28.21)	0.07	0.799

### 3.2 Comparison of EQ-5D-3L scores between before and after intervention

As shown in Table 2, after 12 weeks of telenursing, for the experimental group, each dimension improved, especially for “pain/discomfort” and “anxiety/depression” (*p* < 0.05). Additionally, after the intervention, compared with the control group, all dimensions of the experimental group were better, especially for “pain/discomfort” and “anxiety/depression” (*p* < 0.05). In addition, after 12 weeks of telenursing, for the experimental group, HRQoL and VAS both improved, compared with those before the intervention (*p* < 0.05), and compared with the control group after the intervention (*p* < 0.05).

**Table 2:** Comparison of EQ-5D-3L scores between before and after intervention.

		Experimental group N(%)	Control group N(%)	Z/*t*	*p*
Mobility					
Before intervention	No problems	19 (48.72)	18 (46.15)	0.14	0.887
	Moderate problems	15 (38.46)	13 (33.33)		
	Severe problems	5 (12.82)	8 (20.51)		
After intervention	No problems	21 (53.85)	21 (53.85)	0.50	0.618
	Moderate problems	13 (33.33)	12 (30.77)		
	Severe problems	5 (12.82)	6 (15.38)		
Z		0.65	0.32		
*p*		0.519	0.747		
Self-care					
Before intervention	No problems	13 (33.33)	13 (33.33)	0.51	0.609
	Moderate problems	20 (51.28)	19 (48.72)		
	Severe problems	6 (15.38)	7 (17.95)		
After intervention	No problems	16 (41.03)	12 (30.77)	0.10	0.920
	Moderate problems	16 (41.03)	20 (51.28)		
	Severe problems	7 (17.95)	7 (17.95)		
Z		0.33	0.77		
*p*		0.741	0.444		
Usual Activities					
Before intervention	No problems	13 (33.33)	13 (33.33)	0.14	0.887
	Moderate problems	20 (51.28)	19 (48.72)		
	Severe problems	6 (15.38)	7 (17.95)		
After intervention	No problems	16 (41.03)	12 (30.77)	0.69	0.488
	Moderate problems	16 (41.03)	20 (51.28)		
	Severe problems	7 (17.95)	7 (17.95)		
Z		0.38	0.09		
*p*		0.702	0.925		
Pain/Discomfort					
Before intervention	No problems	4 (10.26)	5 (12.82)	0.53	0.594
	Moderate problems	21 (53.85)	22 (56.41)		
	Severe problems	14 (35.90)	12 (30.77)		
After intervention	No problems	13 (33.33)	6 (15.38)	2.13	0.034
	Moderate problems	20 (51.28)	21 (53.85)		
	Severe problems	6 (15.38)	12 (30.77)		
Z		2.68	0.20		
*p*		0.007	0.84		
Anxiety/Depression					
Before intervention	No problems	6 (15.38)	6 (15.38)	0.36	0.716
	Moderate problems	21 (53.85)	19 (48.72)		
	Severe problems	12 (30.77)	14 (35.90)		
After intervention	No problems	19 (48.72)	7 (17.95)	2.95	0.003
	Moderate problems	15 (38.46)	20 (51.28)		
	Severe problems	5 (38.46)	12 (30.77)		
Z		2.66	0.42		
*p*		0.008	0.674		
HRQoL					
Before intervention		0.56 ± 0.16	0.55 ± 0.13	0.46	0.648
After intervention		0.66 ± 0.19	0.57 ± 0.12	2.52	0.014
*t*		2.62	1.09		
*p*		0.013	0.282		
VAS					
Before intervention		60.26 ± 12.92	56.41 ± 14.64	1.23	0.223
After intervention		73.67 ± 12.80	56.92 ± 10.04	6.43	0.000
*t*		4.38	0.20		
*p*		0.000	0.843		

### 3.3 Comparison of SF-36 scores between before and after intervention

As shown in Table 3, after the intervention, for the experimental group, each dimension score improved, especially for the scores of GH, BP, RE, MH, VT, SF, PCS, MCS, and SF-36, which were statistically significant compared with those before the intervention (*p* < 0.05).

**Table 3:** Comparison of SF-36 scores between before and after intervention.

		Experimental group	Control group	Z/*t*	*p*
GH	Before intervention	61.54 ± 20.62	61.92 ± 20.05	0.08	0.934
	After intervention	63.33 ± 18.76	62.31 ± 19.53	0.24	0.814
	*t*	2.77	1.14		
	*p*	0.009	0.262		
PF	Before intervention	61.92 ± 19.35	60.90 ± 16.66	0.25	0.803
	After intervention	63.46 ± 20.49	61.03 ± 15.82	0.59	0.559
	*t*	1.41	0.22		
	*p*	0.166	0.831		
RP	Before intervention	50.00,50.00/75.00	50.00,50.00/75.00	0.06	0.950
	After intervention	50.00,50.00/75.00	50.00,50.00/75.00	0.26	0.796
	Z	0.82	0.00		
	*p*	0.414	1.000		
BP	Before intervention	52.05 ± 24.20	52.26 ± 24.37	0.04	0.970
	After intervention	56.59 ± 22.40	52.46 ± 24.41	0.78	0.439
	*t*	4.01	0.22		
	*p*	0.000	0.825		
RE	Before intervention	66.67,33.33/66.67	66.67,33.33/66.67	0.02	0.987
	After intervention	66.67,33.33/66.67	66.67,33.33/66.67	1.18	0.239
	Z	2.27	0.45		
	*p*	0.023	0.655		
MH	Before intervention	50.77 ± 21.35	50.05 ± 20.92	0.15	0.881
	After intervention	52.31 ± 22.37	50.56 ± 20.63	0.36	0.721
	*t*	2.31	1.30		
	*p*	0.027	0.201		
VT	Before intervention	61.79 ± 20.79	61.92 ± 20.31	0.03	0.978
	After intervention	63.46 ± 19.17	62.18 ± 19.29	0.29	0.769
	*t*	2.70	0.70		
	*p*	0.010	0.487		
SF	Before intervention	52.88 ± 23.38	53.21 ± 20.22	0.00	1.000
	After intervention	55.13 ± 23.77	53.53 ± 20.06	0.39	0.696
	*t*	2.48	0.33		
	*p*	0.018	0.744		
PCS	Before intervention	58.14 ± 13.12	58.03 ± 12.89	0.04	0.971
	After intervention	60.43 ± 12.32	58.21 ± 12.64	0.79	0.435
	*t*	4.47	0.31		
	*p*	0.000	0.759		
MCS	Before intervention	55.12 ± 16.45	54.97 ± 12.88	0.04	0.965
	After intervention	57.76 ± 16.21	55.03 ± 12.61	0.83	0.409
	*t*	4.48	0.10		
	*p*	0.000	0.918		
SF-36	Before intervention	56.63 ± 13.12	56.50 ± 10.68	0.05	0.962
	After intervention	59.10 ± 12.83	56.62 ± 10.93	0.92	0.362
	*t*	6.25	0.31		
	*p*	0.000	0.755		

### 3.4 Comparison of blood pressure and blood glucose between before and after intervention

**Figure 2:**
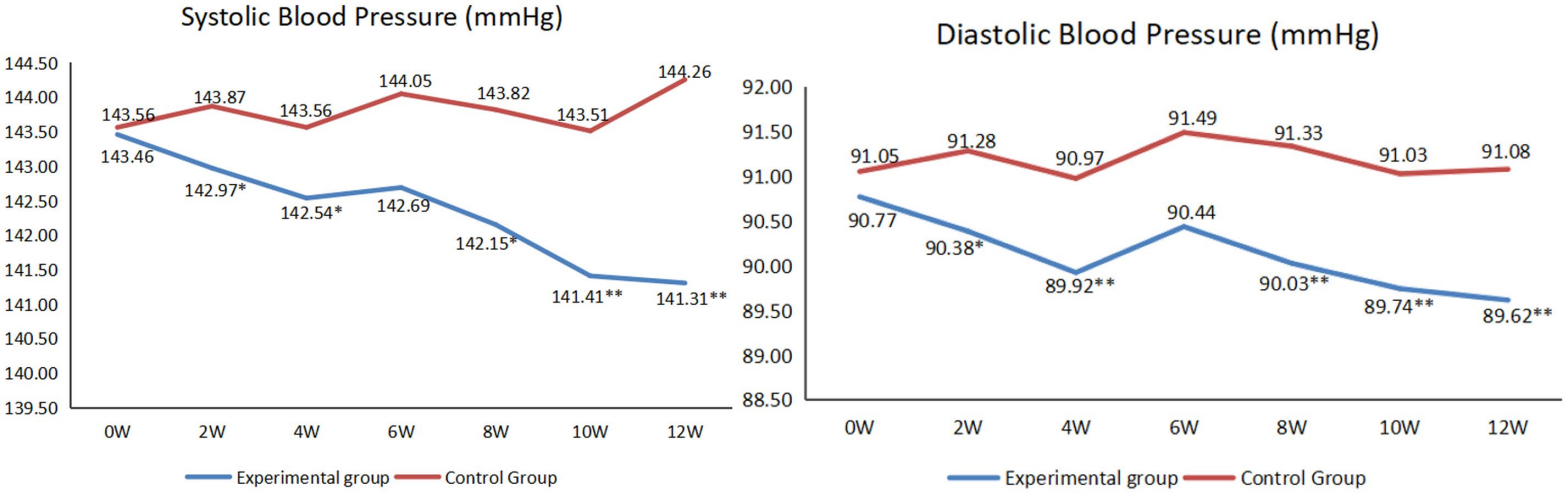
Comparison of systolic and diastolic blood pressure between before and after intervention (*p < 0.05, **p < 0.01).

As shown in Figure 2, for the experimental group, at the end of the 2nd, 4th, 8th, 10th and 12th weeks of the intervention, there were statistically significant in systolic blood pressure (*p* < 0.05) and diastolic blood pressure (*p* < 0.05) compared with those before the intervention.

**Figure 3:**
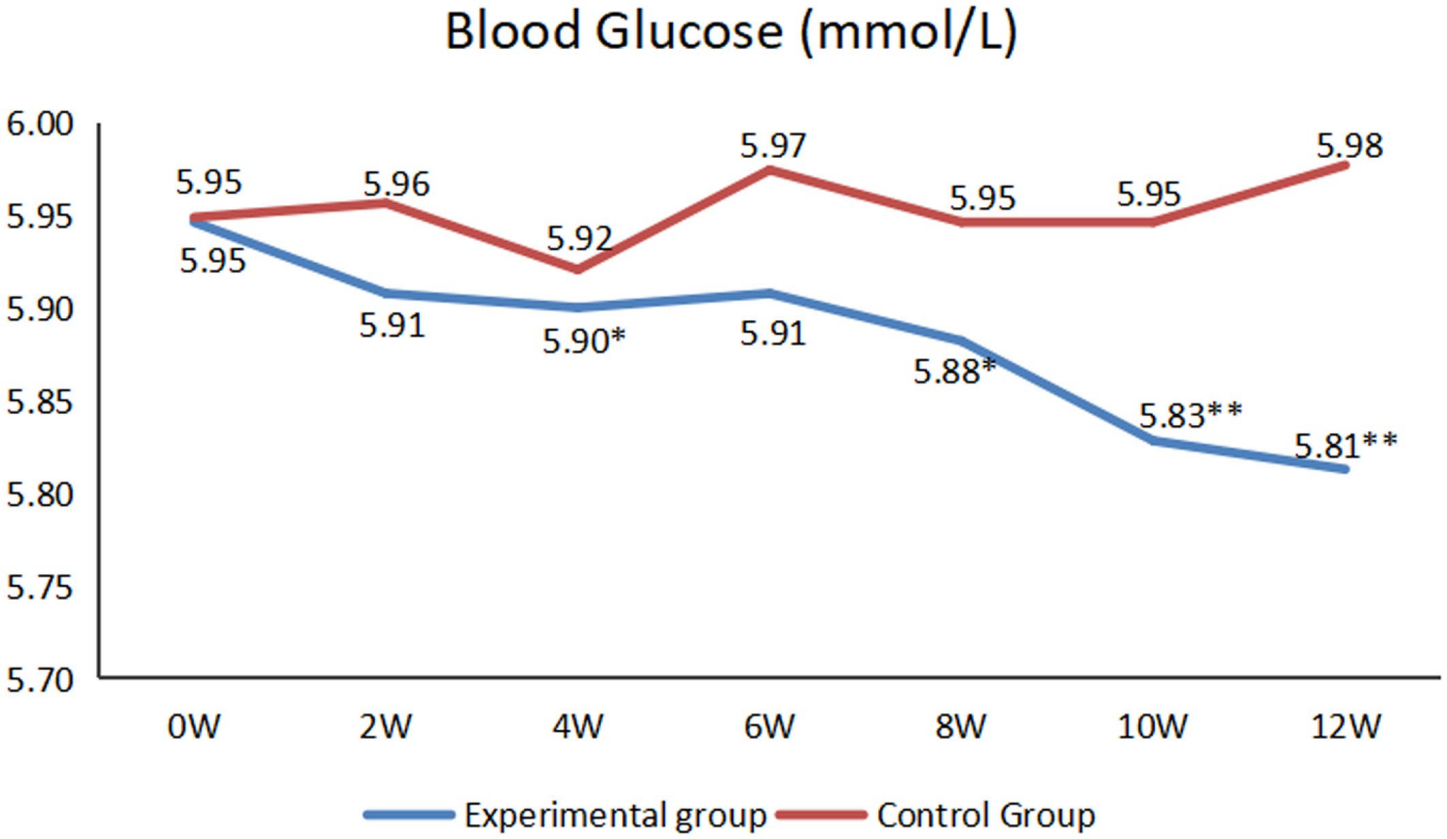
Comparison of blood glucose between before and after intervention (*p < 0.05, **p < 0.01).

As shown in Figure 3, for the experimental group, at the end of the 4th, 8th, 10th and 12th weeks of the intervention, there were statistically significant in blood glucose levels compared with those before the intervention (*p* < 0.05).

### 3.5 Comparison of weight, BMI, Fat rate, water rate, protein rate, and basal metabolic rate between before and after intervention

**Figure 4:**
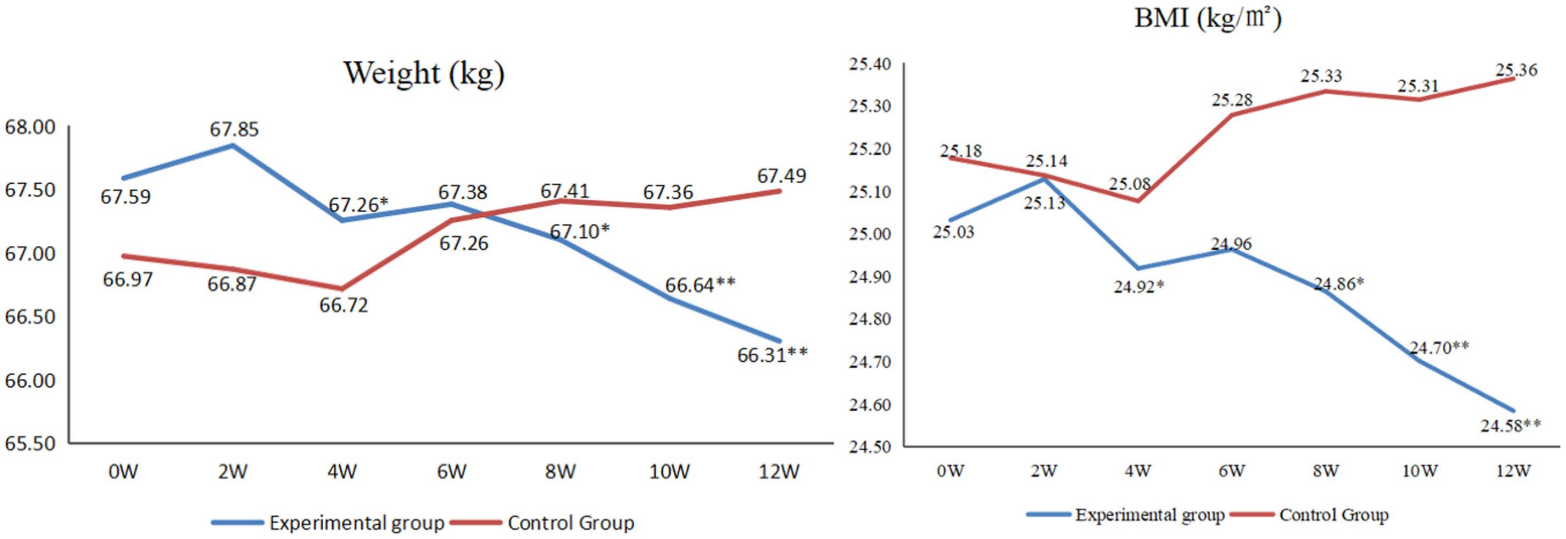
Comparison of weight and BMI between before and after intervention (*p < 0.05, **p < 0.01).

As shown in Figure 4, for the experimental group, at the end of the 4th, 8th, 10th and 12th weeks of the intervention, there were statistically significant in weight (*p* < 0.05) and BMI (*p* < 0.05) compared with those before the intervention.

As shown in Table 4, after the intervention, for the experimental group, each rate improved, especially for the fat rate, water rate and basal metabolic rate, which were statistically significant compared with those before the intervention (*p* < 0.05). Additionally, after the intervention, compared with the control group, researchers found that each rate of the experimental group was better, especially for the fat rate, which was statistically lower than the control group (*p* < 0.05).

**Table 4:** Comparison of fat rate, water rate, protein rate, and basal metabolic rate between before and after intervention.

		Experimental group	Control group	*t*/Z	*p*
Fat rate (%)	Before intervention	32.59 ± 4.20	32.92 ± 4.01	0.36	0.721
	After intervention	31.74 ± 3.60	33.31 ± 3.16	2.04	0.045
	*t*	2.85	1.27		
	*p*	0.007	0.211		
Water rate (%)	Before intervention	48.00,46.00/50.00	48.00,47.00/51.00	0.30	0.972
	After intervention	49.00,48.00/51.00	49.00,47.00/51.00	0.76	0.331
	Z	2.87	0.00		
	*p*	0.004	1.000		
Protein rate (%)	Before intervention	14.92 ± 3.78	14.41 ± 2.85	0.63	0.501
	After intervention	15.03 ± 3.41	14.03 ± 2.75	1.43	0.158
	*t*	0.57	1.92		
	*p*	0.570	0.062		
Basal metabolic rate (kcal/d)	Before intervention	1272.51 ± 141.11	1278.67 ± 134.73	0.20	0.844
	After intervention	1291.59 ± 136.90	1277.15 ± 135.68	0.47	0.641
	*t*	3.18	0.55		
	*p*	0.003	0.587		

### 3.6 Comparison of sleep condition between before and after intervention

**Figure 5:**
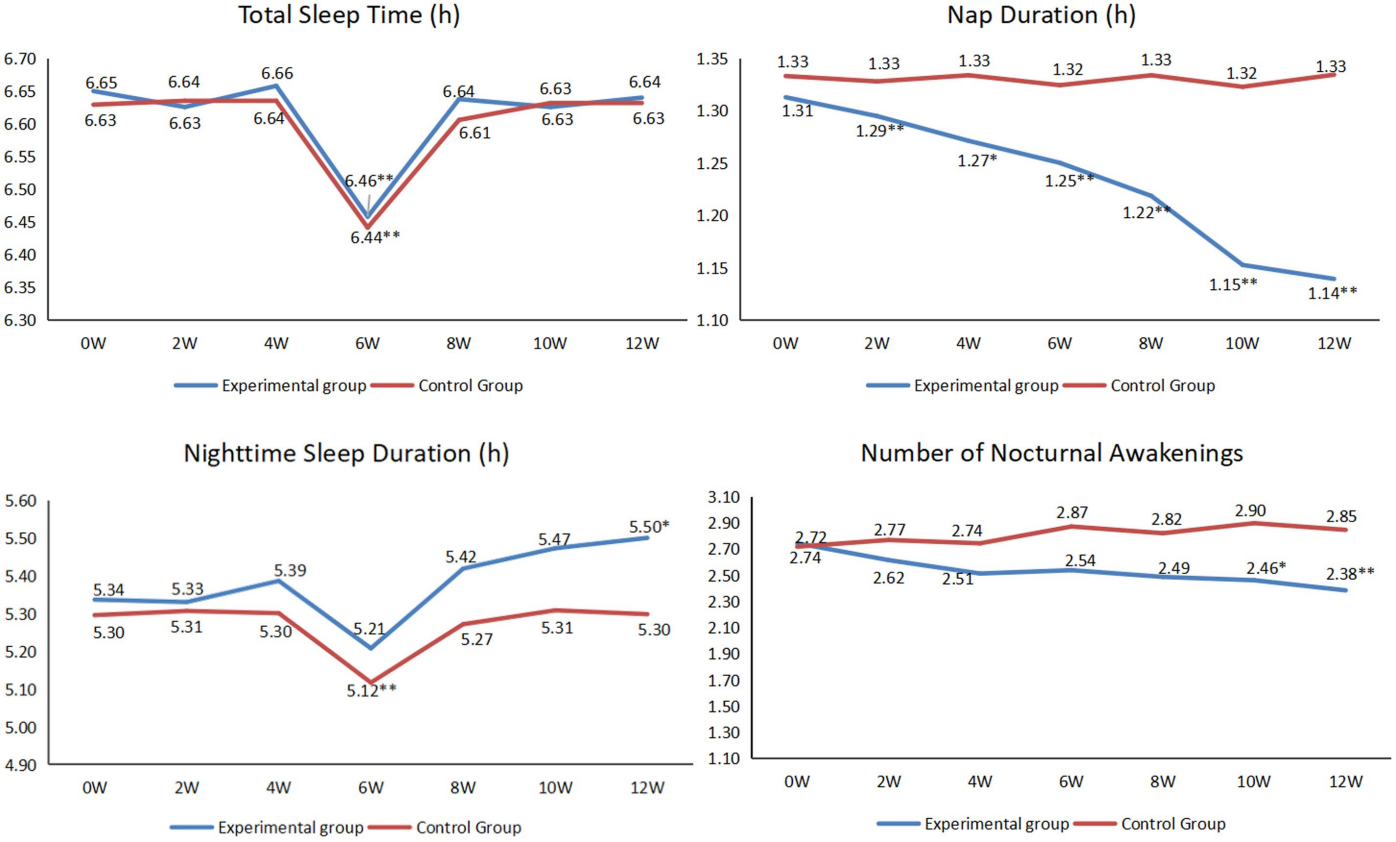
Comparison of the sleep between before and after intervention (*p < 0.05, **p < 0.01).

As shown in Figure 5, for the experimental group, there were no statistically significant in total sleep time throughout the intervention except at the 6th week of the intervention (*p* < 0.05). From the beginning of the intervention, the nap duration began to decrease, at the end of the 2nd, 4th, 6th, 8th, 10th and 12th weeks of the intervention, there were statistically significant compared with those before the intervention (*p* < 0.05). Except for the 6th week, the overall sleep time at night showed an upward trend, especially at the end of the 12th week (*p* < 0.05). The overall number of nocturnal awakenings showed a downward trend, especially at the end of the 10th and 12th weeks (*p* < 0.05).

**Figure 6:**
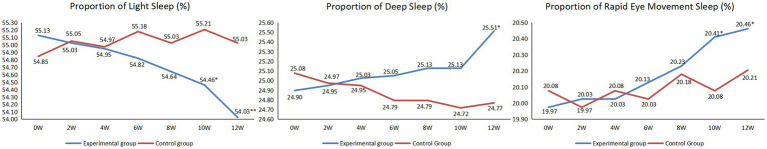
The proportion of light sleep, deep sleep and rapid eye movement sleep between before and after intervention (*p < 0.05, **p < 0.01).

As shown in Figure 6, for the experimental group, the overall light sleep rate showed a downward trend, especially at the end of the 10th and 12th weeks (*p* < 0.05). While the overall deep sleep rate showed an upward trend, especially at the end of the 12th week (*p* < 0.05). Additionally, the overall rapid eye movement sleep rate showed an upward trend, especially at the end of the 10th and 12th weeks (*p* < 0.05).

## 4 Discussion

### 4.1 M-O-A telenursing intervention can effectively improve quality of life of the empty-nest older adult individuals with chronic diseases

EQ-5D-3L indicated that, the quality of life of the empty-nest older adult individuals with chronic diseases was poor and “anxiety/depression” was the most troubling thing for them. Through qualitative interviews, it was found that there were four main factors affecting anxiety and depression: economy, company, knowledge and COVID-19. M-O-A telenursing intervention relieved anxiety and depression considerably by the 4 aspects. First, most chronic diseases brought a great economic burden to individuals and families to make them more prone to anxiety and depression (24), Because this intervention was free, it alleviated some of their economic pressure compared with going to the hospital. Second, researchers communicated remotely with the older adult individuals almost every day and visited them at home every 2 weeks. Previous studies discovered that companionship was the best way to alleviate abnormal emotions (25). As for knowledge, researchers provided remote health guidance and disease prevention for the empty nesters to make them know more about chronic diseases and build their self-confidence in fighting with diseases, this is consistent with previous studies (26). Regarding COVID-19, previous studies have demonstrated that a large amount of COVID-19 information made people confused between true and false, which was the a cause of anxiety and depression (27). In this study, researchers provided them with official and real information and helped them deal with some problems that could not be solved because of isolation, such as buying drugs. All these telenursing interventions made them feel warmer and more at ease. EQ-5D-3L also indicated that the second prominent problem for empty-nest older adult individuals patients with chronic diseases was “pain/discomfort.” Previous study have shown that many patients with chronic diseases suffer from pain (28). After M-O-A telenursing intervention, the patients’ pain was relieved statistically. The main reasons were as follows: (a) In the process of telenursing intervention, patients were taught some pain relief methods (29). As a result, patients had a better understanding of their own diseases, established good living and working habits, and controlled or slowed down the development of pain (30). (b) Although “pain/discomfort” was a physiological index, it would be affected by psychological factors. Therefore, when patients’ anxiety and depression were relieved, their “pain/discomfort” was also relieved, which was consistent with previous studies (31).

SF-36 questionnaire showed that, for the experimental group, each dimension score improved. All psychological-related indicators (RE, MH, VT and SF) improved statistically. In addition, although they were physiological-related indicators, the differences in GH and BP, which were greatly influenced by psychology, were also statistically significant, which was consistent with the results of the EQ-5D-3L questionnaire. This further proved that M-O-A telenursing could effectively relieve physical pain and improve the mental health of patients, this was consistent with previous studies (32). PF means “Physical Functioning” and RP means “Physical Role” (33). Although there were no statistically significant, the two indicators also improved in numerical terms. The main reason might be that the improvement of physiological indicators required more time than psychological indicators. In addition, for empty-nest older adult individuals with chronic diseases, due to the increase in age and the consumption of chronic diseases, the physiological indicators inevitably worsened. However, through telenursing, patients could learn more about disease prevention and nursing, could be diagnosed with diseases in time, and could be sent to the hospital in time in case of sudden diseases. Some studies have found that not all telenursing is superior to routine nursing, the main reason is the lack of effective assessment (17), in this study, the existence of these intervention measures based on a thorough needs evaluation delayed the deterioration of patients’ psychological and physiological indicators to a certain extent and improved their quality of life effectively.

### 4.2 M-O-A telenursing intervention can regulate the blood pressure and blood glucose of empty-nest older adult individuals with chronic diseases

Hypertension is a widespread and severe global public health issue (34). Older adult individuals often have a high prevalence of hypertension (35). During the intervention, researchers taught the hypertensive patients how to adjust their blood pressure from the aspects of diet, exercise and rest through remote lectures, video calls and so on. Additionally, because the researchers contacted the patients almost every day, the researcher acted as a supervisor, they supervised patients to take measures to control their blood pressure. Therefore, from the second week, the blood pressure of the experimental group decreased statistically. However, at the end of the sixth week, both systolic and diastolic blood pressure increased. This was mainly because this period of time was China’s Spring Festival. Generally, their fat intake increased, and they had less exercise and sleep (36). For the experimental group, after Spring Festival, researchers quickly adjusted the patients’ lifestyle through remote intervention, and the blood pressure gradually returned to the level before the Spring Festival. Through telenursing, we can enable patients to have a healthy lifestyle. Even if there are occasional eating or sleeping disorders, we can also guide them to adjust quickly and enjoy life healthily. Similarly, for blood glucose, the experimental group as a whole showed a downward trend. Although there was an increase at the end of the sixth week of the intervention, it was not statistically significant. Then, it can be seen at the 10th and 12th weeks of intervention, the data tended to be flat. This was mainly because, for the experimental group, the researchers intervened with the patients through remote lectures, video calls and other forms so that the participants’ blood glucose showed a downward trend and tended to be stable (37).

### 4.3 M-O-A telenursing intervention can regulate weight, BMI, Fat rate, water rate, protein rate, and basal metabolic rate of empty-nest older adult individuals with chronic diseases

Regarding weight, it showed a downward trend from the fourth week, except for the sixth week [similar to the change in blood pressure, this change was mainly due to the rich diet and the reduction in exercise and sleep during the Spring Festival, in line with previous results (38)]. The change trend of BMI was completely consistent with that of body weight. Previous studies have shown that in China, mean BMI and obesity in adults have increased steadily since the early 1980s (39). Obesity is a major risk factor for cardiometabolic diseases and many other diseases (40). Therefore, we need to take active measures to reduce the weight and BMI of residents. Through this study, we found that M-O-A telenursing was a good intervention. We guided patients’ lifestyle from all aspects. For example, if the patient continued to sit for an hour, the smart bracelet would send a “sedentary reminder.” In addition, in the WeChat group, participants urged each other to exercise. Moreover, researchers informed different patients of different types of exercise intensity and action choices through remote lectures. All these interventions made patients thinner and healthier.

Additionally, the percentage of body fat in the experimental group decreased statistically, the percentage of body water and basic metabolic rate increased statistically, and the percentage of body protein increased although there was no statistically significant over time. For adults, the basal metabolic rate will decrease with the loss of skeletal muscle as they grow older (41). Aerobic exercise can improve the basic metabolic rate to achieve the improvement of body composition (42) and it can prevent osteoporosis, enhance the functions of respiratory and circulatory systems, and regulate psychological and mental status. Researchers taught participants Baduanjin, Tai Chi, square dance and so on through recorded videos (43–45), and then, corrected their problems through video calls family visit every 2 weeks. Compared to some previous telenursing methods (46), this study has included some exercises with national characteristics. This kind of exercise was rated favorably by the participants and enabled them to improve their body composition and basic metabolic rate in the process of exercise.

### 4.4 M-O-A telenursing intervention can improve sleep condition of empty-nest older adult individuals with chronic diseases

Sleep health has important consequences for aging trajectories, and sleep disruption has been associated with an increased risk of depression, cognitive decline, Alzheimer’s disease (AD), and cardiovascular and metabolic outcomes (47, 48). First, a previous study found that too much nap duration increased the risk of AD (49). In this study, the nap duration of the experimental group decreased statistically from the second week. This is mainly because, at the beginning of the intervention, the researchers emphasized the harm of excessive nap duration to the patients, and continued to monitor and remind during the whole intervention process. Focusing on cognitive decline, both short sleep (≤6 h/night) and long sleep (≥9 h/night) durations have been associated with worse outcomes (50). In this study, the sleep time at night of the two groups was obviously insufficient. However, through the 12-week intervention, the whole process of the experimental group showed an upward trend. Therefore, we believe that the nighttime sleep duration in the experimental group will become normal in the near future. Regarding the number of nocturnal awakenings, the patients in the experimental group showed a downward trend, especially at 10 weeks and 12 weeks. The main reason was that researchers gave some remote guidance to patients’ sleep, such as reducing nap duration, improving sleep environment, changing eating habits, adjusting living habits, paying attention to moderate exercise, or taking a bath before going to bed. Therefore, the overall sleep quality increased.

Some previous studies have also focused on the improvement of patients’ sleep quality through telenursing (51). This study not only focused on the overall sleep quality of patients, but also on sleep rate. Light sleep is a normal physiological need. However, if the proportion of light sleep in the total sleep time is too high, the sleep quality will become worse (52). Research has found that in the deep sleep stage, the brain can get enough rest, and the effect of eliminating fatigue is also the best. Generally, the longer the deep sleep is, the better the sleep quality (53). In the rapid eye movement sleep stage, muscles are generally paralyzed. This is a safety protection mechanism to prevent us from taking actions in our dreams (54). Before the start of the intervention, the proportion of light sleep in the two groups was slightly higher. During the intervention, for the experimental group, the proportion of light sleep decreased, and the proportion of deep sleep and rapid eye movement sleep increased. It can be seen M-O-A telenursing intervention can effectively regulate the sleep rate.

## 5 Conclusion

The M-O-A telenursing model could effectively regulate quality of life and health condition of the empty-nest older adult individuals with chronic diseases, making it worthy of further promotion and application.

## Limitations and future studies

This study has a few limitations, and we will try to make this study more meaningful in the future. First, although this study met sample size calculation, whether the results can be generalized to other cities or countries needs further research. In the future, we can continue to expand the research area and sample size to obtain more general conclusions. Second, since the intervention effects of the experimental group were statistically significant by the 12th week of intervention, this study selected the data of these 12 weeks for analysis. In the future, we can continue to carry out M-O-A telenursing intervention to examine the long-term effects.

## Data availability statement

The original contributions presented in the study are included in the article/supplementary material, further inquiries can be directed to the corresponding author.

## Ethics statement

The studies involving humans were approved by Ethics Committee of School of Nursing, Yangzhou University. The studies were conducted in accordance with the local legislation and institutional requirements. The participants provided their written informed consent to participate in this study.

## Author contributions

YY, PH, and JL designed the study protocol. YY, PH, and SW performed the data collection and interpretation. AK and KY performed statistical analysis. YY and PH drafted the manuscript. JL revised the article. All authors contributed to the article and approved the submitted version.
